# BCEDB: a linear B-cell epitopes database for SARS-CoV-2

**DOI:** 10.1093/database/baad065

**Published:** 2023-09-30

**Authors:** Chengzheng Tai, Hongjun Li, Jing Zhang

**Affiliations:** Key Laboratory for Biomechanics and Mechanobiology of Ministry of Education, Beijing Advanced Innovation Centre for Biomedical Engineering, School of Engineering Medicine & School of Biological Science and Medical Engineering, Beihang University, No. 37 Xueyuan Road, Haidian District, Beijing 100191, China; Department of Radiology, Beijing YouAn Hospital, Capital Medical University, No. 8 Youan Gate Outer Xitou Alley, Beijing 100069, China; Key Laboratory for Biomechanics and Mechanobiology of Ministry of Education, Beijing Advanced Innovation Centre for Biomedical Engineering, School of Engineering Medicine & School of Biological Science and Medical Engineering, Beihang University, No. 37 Xueyuan Road, Haidian District, Beijing 100191, China

## Abstract

The 2019 Novel Coronavirus (SARS-CoV-2) has infected millions of people worldwide and caused millions of deaths. The virus has gone numerous mutations to replicate faster, which can overwhelm the immune system of the host. Linear B-cell epitopes are becoming promising in prevention of various deadly infectious diseases, breaking the general idea of their low immunogenicity and partial protection. However, there is still no public repository to host the linear B-cell epitopes for facilitating the development vaccines against SARS-CoV-2. Therefore, we developed BCEDB, a linear B-cell epitopes database specifically designed for hosting, exploring and visualizing linear B-cell epitopes and their features. The database provides a comprehensive repository of computationally predicted linear B-cell epitopes from Spike protein; a systematic annotation of epitopes including sequence, antigenicity score, genomic locations of epitopes, mutations in different virus lineages, mutation sites on the 3D structure of Spike protein and a genome browser to visualize them in an interactive manner. It represents a valuable resource for peptide-based vaccine development.

**Database URL:** http://www.oncoimmunobank.cn/bcedbindex

## Introduction

The novel coronavirus disease 2019 (COVID-19) has spread rapidly around the world. By 11 June 2022, more than 534 millions confirmed cases and more than 6 millions deaths were reported by 212 countries or regions. Many places have experienced the peak of the epidemic repeatedly. The Coronaviridae Study Group (CSG) of the International Committee on Taxonomy of Viruses, which is responsible for developing the virus classification and Coronaviridae classification nomenclature, assessed the placement of the human pathogen, tentatively named 2019-nCoV, within Coronaviridae. According to phylogeny, taxonomy and established practice, CSG believes that the virus forms a sister clade to the prototype of human and bat severe acute respiratory syndrome coronaviruses (SARS-CoVs), and designated it as SARS-CoV-2 (1). Like other coronaviruses, SARS-CoV-2 has a large RNA genome composed of ∼ 30 000 nucleotides, and its replication is mediated by RNA dependent RNA polymerase (RdRP) and related proofreading enzyme exonuclease (ExoN) (2). This is combined with the discontinuity of coronavirus transcription, resulting in high recombination rate, insertion and deletion rate and point mutation rate of the coronavirus (2). One recent mutant strain was named *Omicron* by WHO, which erupts quickly, is highly contagious and difficult to eliminate (3). It causes more than 200 sequelae and brings long-term damage to human body functions and may become the most dangerous SARS-CoV-2 variant of concern (VOC).

Studies have found that SARS-CoV-2 enters cell through the combination of Spike protein and human angiotensin converting enzyme2 (ACE2). The Spike protein includes two subunits, S1 and S2, of which S1 is mainly responsible for the recognition of receptors and mediates the binding of the virus to the host cell. The activation of S2 mediated by the S1 subunit can strengthen the combination between S1 and the host cells, thereby promoting the proliferation of the virus in host cells. The S1 subunit can be further divided into two independent domains, namely the N-terminal domain (NTD) and the C-terminal domain (CTD). There is an important receptor binding domain (RBD) on S1, which determines the binding specificity of the virus to human ACE2 (4).

One likely optimistic scenario is the transition to an epidemic seasonal disease such as influenza; another scenario is that sustained high levels of infection will lead to further evolution of the virus (5). In any case, vaccines are the most important means to achieve herd immunity (6). Polypeptide vaccines are vaccines prepared by polypeptide synthesis technology according to the amino acid sequence of a certain segment of the antigenic epitope known or predicted in the antigenic gene of the pathogen. SARS-CoV-2 mutates rapidly, and there are more than 2.9 million strains, many of which are highly infectious, such as a series of strains named and concerned by WHO. Polypeptide vaccines can be prepared quickly. They have a favorable safety profile and induce potent immune responses after a single vaccination if the appropriate epitope is selected (7). Moreover, the polypeptide backbone is composed of amide bonds, which is more conducive to being taken up by cells (8), so it has become a hot spot of clinical research.

An antigenic epitope is a special chemical group that determines the specificity of the antigen in an antigen molecule, also known as an antigenic determinant. An antigen can specifically bind to a cell surface antigen receptor through an antigenic epitope, thereby causing an immune response (9). Fragments that can be specifically recognized and bound by B-cell surface receptors are B-cell epitopes. Epitopes are target structures recognized by immune cells and the basis of specific immune responses, which is vital in vaccine development. Finding candidate epitopes that may have antigenicity is an important step in epitope identification. Machine learning methods have been widely used in epitope prediction. Bepipred (10) uses a combination of hidden Markov models and propensity scale methods to predict the location of linear B-cell epitopes. Bepipred II (11) incorporates conformational epitopes as a consideration and predicts epitopes by a random forest algorithm. ABCpred server (12–15) predicts epitopes with 65.93% accuracy using a recurrent neural network. Epidope (16) used the ELMo model of language learning to represent the residue context, achieving an area under the receiver operating characteristic curve (AUC) of 0.67. For proteins with structural data files in Protein Data Bank (PDB), ElliPro (17) predicts linear and discontinuous antibody epitopes, achieving an AUC of 0.732. Totally, machine learning methods are facilitating the vaccines’ development by providing predicted epitopes as candidates.

Several databases on SARS-CoV-2 have been released since 2019 (18). RCoV19(https://ngdc.cncb.ac.cn/ncov), part of the China National Center for Bioinformation (CNCB), collected publicly available data on lineages and variants on the Internet, containing a wide range of relevant information, including scientific literature, news and popular articles used for science communication, and provides visualization capabilities for genomic variation analysis results based on all collected SARS-CoV-2 strains. It focuses on the virus itself and clinical data from relevant institutions. Novel Coronavirus Information Center (https://www.elsevier.com/connect/coronavirus-information-center) created a range of free resources, including evidence-based clinical guidance and more than 41 000 research articles to read, download and data mine, and is offering front-line clinical tools and resources to help healthcare professionals deliver the best care and patient education. Cov-Lineages.org (https://cov-lineages.org/) was developed to implement the dynamic nomenclature of SARS-CoV-2 lineages, known as the Pango nomenclature. It documented all current Pango lineages and their spread, providing tools for assigns lineages to sequences, generating a report for a set of sequences, single nucleotide polymorphism (SNP)-based calling of VOCs and getting view and interaction with the latest global SARS-COV-2 phylogenic tree. In addition, academic journals, including JAMA, Lancet, NEJM, BMJ, and gene databases like NCBI (https://www.ncbi.nlm.nih.gov/), UCSC (http://www.genome.ucsc.edu/) all dedicated areas to publish COVID-19-related literature and sequences. Existing databases focused more on upstream data and visualization of existing data.

Research has shown that mutations in the viral spike protein affect its ability to infect host cells and to evade host immunity. The acquisition of mutations at the site where the spike protein epitope is located is essential for the preparation of peptide vaccines. Here, we develop BCEDB database, which concentrates on epitopes predicted from Spike protein sequence and epitopes mined from literature, to facilitate the development of polypeptide vaccines and compensate for the absence of the immune epitope database of SARS-CoV-2. BCEDB database also collect lineages sources and map them to spike protein mutations and epitopes, respectively. Additionally, this paper introduces the implementation method and use cases of the BCEDB database.

## Methods

### Data collection

The primary sequence of SARS-CoV-2 and all its variants of Spike protein were retrieved from NGDC (19–22) and NCBI (https://www.ncbi.nlm.nih.gov/). 3D structure of SARS-CoV-2 Spike protein (PDB ID: 6VSB) was retrieved from Protein Data Bank (PDB). The lineage-related data of SARS-CoV-2 was retrieved from Cov-Lineages.org (23).

### Epitopes through literature mining

We integrated published literature about epitope predictions. We collected 17 articles (24–40) and obtained 248 B-cell epitopes. We used Blastp to locate each epitope and these positions were used to evaluate the combining ability with human ACE2. Epitopes located in the RBD region were considered as potential epitopes and would be further evaluated. We submitted these potential epitopes to VaxiJen v.2.0 Server (41) to analyze the antigenicity of epitopes. We evaluated allergenicity of B-cell epitopes by Allergen FP 1.0 (42) and assessed toxicity, hydrophobicity, hydropathicity, hydrophilicity and charge by ToxinPred (43).

### Epitopes through prediction

We used B-cell epitope Prediction tools including ABCPred (12), BCPred, AAP, FBCPRED (13–15), ellipro (17) and Emini Surface Accessibility Prediction, Kolaskar & Tongaonkar Antigenicity, Bepipred Linear Epitope Prediction and Bepipred Linear Epitope Prediction2.0 methods from the immune epitope database (IEDB) (44), with default parameters. The results were merged into the linear B-cell epitope candidate list for further screening. We predicted the transmembrane topology of Spike protein by TMHMM v2.0 (https://dtu.biolib.com/DeepTMHMM), retained the epitopes on the outer surface and removed other intracellular epitopes. A total of 3720 epitopes remained. Then antigenicity of epitopes was evaluated by VaxiJen V2.0 (41), and hydrophilicity, hydrophobicity, charge, sensitization and toxicity of epitopes were further assessed by allergens FP1.0 (42) and ToxinPhred (43), respectively (Table 1).

**Table 1.** BCEDB data content and statistics

		Number of features						
Type	Tools	Total	Antigenicity > 0.4	Antigenicity > 0.6	Non-allergenic	Non-toxin	Hydropathicity > 0	Topology_outside
B cell epitopes	Total	60 768	33 968	22 752	39 440	58 816	22 784	59 232
	BCPRED	2 960	1 440	880	2 176	2 896	608	2 880
	ABCPRED	48 112	26 896	17 744	34 704	46 448	19 472	39 872
	FBCPRED	3 440	1 776	1 056	2 624	3 264	1 104	3 296
	IEDB	4 080	2 272	1 664	1 968	4 032	880	3 952
	Litarature	3 968	2 464	1 936	2 480	3 936	1 280	3 872
	AAP	3 056	1 776	1 120	2 176	2 944	816	2 960
	COVIDep	432	256	192	304	432	224	400
	ellipro	32	21	15	20	32	8	32
Spike protein variants	-	Total	9 793	ACE2	6 503	Key interactions	742	
Lineage	-	Total	37 602	WHO named	438	Date& description	7 584	

### System architecture

**Figure 1.**
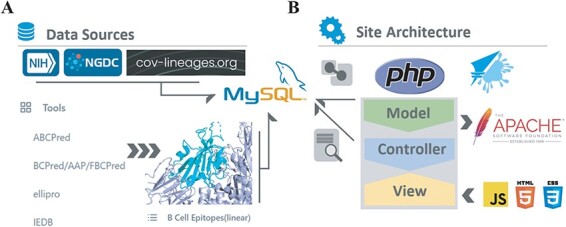
System architecture. (A) B-cell epitopes predicted by ABCPred, BCPred, AAP, FBCPred, ellipro and IEDB. Spike protein variants and lineages data form NIH, NGDC and cov-lineages, all stored in MySQL server. (B) Using MVC framework to fetch data items.

Like existing databases (45, 46), BCEDB utilized a DIV layout, browser/server architecture and used MySQL (http://www. mysql. org) for data storage. MySQL is a relational database management system. Lineage data including number of samples, description, date of earliest report, variants and lineage distribution, B-cell epitopes including its sites, peptide length, antigenicity and other properties and Spike protein mutations were stored in the database as tables. The Web interface was mainly based on Html, CSS and JavaScript. BCEDB adopts MVC architecture. The MVC (Model, Controller, View) architecture, based on PHP, improves the maintainability, portability, scalability and reusability of the software (Figure 1). The controller was responsible for receiving user requests, sending the instructions and requests input by the user to model, receiving the data returned by model and calling the corresponding view to return the results to the user. We stored all the database connections and query functions in model. Our model used the singleton pattern, which ensured that the class only ran one instance every time, to provide a global point of access to the system as a whole, to save memory space, to avoid frequent create destruction of objects, to improve performance and to simplify the access. Views were primarily based on the Bootstrap open-source framework, enhancing the visibility and usability of our resources. Our site used the Apache2 (http://httpd.Apache.org/) HTTP server on the Ubuntu Linux platform.

## Results

### B-cell epitopes browsing

**Figure 2.**
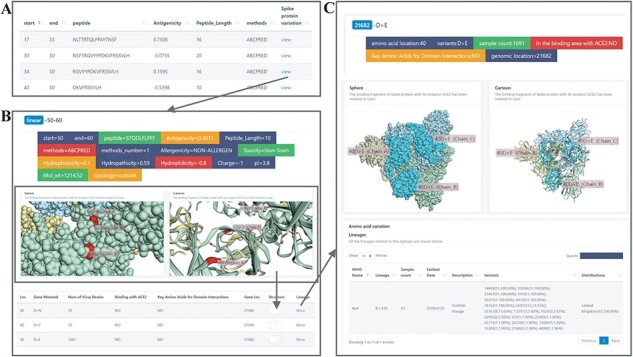
B-cell epitopes browsing. (A) B-cell epitopes with its antigenicity. Click the ‘view’ button to view the Spike protein mutations. (B) Spike protein mutations related. View the mutation sites by switching and view lineages by button ‘view’. (C) Lineages related with Spike protein structure.

This module lists all the B-cell linear epitopes of SARS-CoV-2 (Length ≥3), and provides properties associated with the epitopes including antigenicity, length and prediction methods of the epitope, and a link to browse specific information about the epitopes, including other properties of the epitope and Spike protein mutation data associated (Figure 2). We use 3Dmol.Js (http://3dmol.csb. pitt.edu/) to visualize the structures in cartoon and sphere, respectively. Spike protein of SARS-CoV-2 is a trimer and we use different light colors to distinguish between them, and mark area combined with human ACE2 Cyan. Users can select the Spike protein variants which they are interested in and highlight them in red on the structure diagram. This function allows the simultaneous labeling of multiple Spike protein variants. In the table of Spike protein variation data, we provide a link to browse the specific information of the mutation and the lineages associated with the mutation site. Users can also find the Spike protein structure diagram with the mutation location marked here.

### Lineages browsing

**Figure 3.**
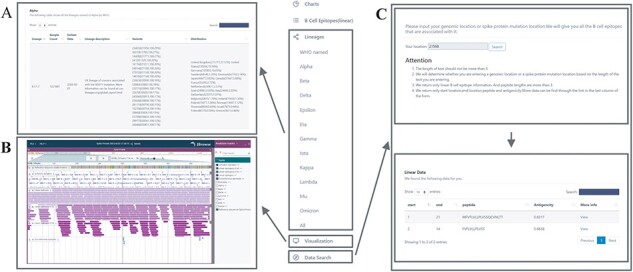
Important functions of database. (A) Lineages browse, an example of WHO named lineage. (B) Jbrowse genome browser for visualization. (C) Search function; click the ‘view’ button to view details.

The Pango naming method is based on phylogenetic structure, which clearly indicates where the mutant is in evolution and how closely related it is to other mutants. Via studying lineages, researchers can get a better handle on how the virus mutates. We collected data of the COVID-19 lineages with special emphasis on lineages named by WHO, which you can easily find in the sidebar. Users can easily find them to get a macro view of virus variation or can explore related lineages through B-cell epitopes, which are associated with lineage through gene location information. Browsing S-protein variants also provide a link to related lineages. Features including Pango name, sample count, variants data, sequence distribution, discovery date and lineage introduction are provided (Figure 3A).

### Visualization

Genome browser is an important visualization tool for high-throughput sequencing analysis. It can provide more information than the final tables provided. Jbrowse (https://www.jbrowse.org/jb2/) is a genome browser with good compatibility; we embed Jbrwose2 in the view, with Spike protein of SARS-CoV-2 as reference sequence, including the visualization of RBD, NTD, Spike protein variation loci, B-cell epitopes and Spike protein mutation information of lineages that WHO is concerned about (Bigure 3B). We believe that epitopes with an antigenicity score of 0.9 are adequate for defensive immune response, and an antigenicity score of 0.4 is also noteworthy, so we screened linear B-cell epitopes with antigenicity >0.4 and antigenicity >0.9 for browsing. We also provide all discontinuous B-cell epitopes here. They can be reorganized for personalized use.

### Search and epitope prediction tools

We provide epitope search function. By entering a genomic location or a Spike protein mutation location, our site will return all related B-cell linear epitope information (Figure 3C) and an entrance for detailed information. Epitope prediction is suitable for the prediction of linear epitopes of proteins or polypeptide antigens with known primary structure. The prediction tools we used are put together: ABCPred, BCPred, AAP, FBCPRED, ellipro and IEDB, and their introductions and links are provided. The prediction methods we use are all machine learning methods based on neural networks. IEDB is a collection of methods to predict linear B cell epitopes based on sequence characteristics of the antigen using amino acid scales and Hidden Markov Model (HMM). Links to datasets used to train the neural networks are also available.

### Data download and help

Help page provides a guide to the use of the website. It gives an introduction to the main content of the website. Entrance to help is also placed on home page. The structure of web site data browsing is presented in the form of a tree branch diagram so that users can find how they can access the content they want. Download links for all our tables are available at the bottom of help page.

### The utility of BECDB

Case study 1: Li W, *et al.* carried out a prediction work on S-protein-based epitopes against SARS-COV-2 through literature mining (47). Considering that various screening and verification methods were applied to obtain epitopes in each work, those predicted epitopes are not entirely consistent, lacking in evidence from *in vitro* and *in vivo* experiments. They subsequently found that linear B-cell epitopes predicted in multiple studies converged to three hot spots in the S-protein RBD. The three hotspot regions harbored three B-cell linear epitopes including ‘RQIAPGQTGKIADYNYKLPD’, ‘SYGFQPTNGVGYQ’ and ‘YAWNRKRISNCVA’. They examined the locations of the three B-cell linear epitopes on Spike protein through 3D structures provided by both BCEDB database and pymol. The three epitopes were consistently found on the exposed region of the Spike protein. They also compared the toxicity, hydrophilicity, hydrophobicity and charge of the three epitopes with those documented in BCEDB and determined that they had high potential for vaccine development.

Case study 2: Li L, *et al.* adopted an immune-informatics-based pipeline with highly stringent criteria to identify S, M and N protein targeted B- and T-cell epitopes that may potentially promote an immune response in the host (48). They preliminarily examined the locations of the predicted B-cell linear epitopes on Spike protein using 3D structure provided by BCEDB database, which were parallelly confirmed by pymol. They finally found 10 valuable B-cell epitopes in the exposed region of Spike region. They additionally assessed whether the predicted epitopes contained any mutations in different lineages in BCEDB, which were then consistently verified in NGDC. Five of the 10 B-cell epitopes were found containing no mutations and were believed to have high potential for vaccine development.

## Discussion

The outbreak of the SARS-CoV-2 has infected millions of people and is still spreading all over the world. There are no effective treatment and prophylaxis methods yet. Polypeptide vaccine with tremendous potential is considered as a possible means to end the epidemic. We obtained 248 B-cell epitopes through mining the literature and 3720 B-cell epitopes through multiple prediction tools and developed BCEDB database to display linear B-cell epitopes and their properties. We obtained a total of 3836 epitopes, 2154 epitopes with an antigenicity score >0.4 and 640 epitopes with an antigenicity score >0.9. Among the epitopes with an antigenicity score greater than 0.9, 398 epitopes showed neither sensitization nor toxicity, indicating high potential for vaccine production. Compared with existing databases, the BCEDB database focuses on B-cell epitopes of SARS-CoV-2 rather than strain variants. We integrate relevant information of B-cell epitopes, and provide multiple visualizations. In addition, we mapped the variation and lineage data of Spike protein to B-cell epitopes, enabling users to have a more specific understanding of the epitopes. BCEDB database compensates for the absence of the immune epitope database and helps to determine appropriate epitopes via comprehensive epitope features and associated sequence mutations to providing reference for polypeptide vaccine for SARS-CoV-2. Users can also get a reference from the database for epitope prediction. The SARS-CoV-2 is still in a state of flux, so we will be updating our data to keep our S-protein variation data and pedigree data up-to-date. And we will continue to add new epitope prediction tools to expand the database. Although we have integrated multiple prediction methods to obtain more informative results, antigenicity scores still cannot fully reflect the true antigenicity of epitopes. *In vitro* validation (such as using organoid) (49, 50) and neutralization assay (51) may be helpful to confirm the antigenicity of predicted epitopes. In the future, we will study more accurate epitope prediction methods and apply them to a wider range of viruses.

## Data Availability

BCEDB is available at http://www.oncoimmunobank.cn/bcedbindex, where all data can be downloaded.
